# Complete mitochondrial genome of the Satanas beetle, *Dynastes satanas* Moser, 1909 (Coleoptera: Scarabaeidae)

**DOI:** 10.1080/23802359.2024.2432373

**Published:** 2024-12-01

**Authors:** Xunuo He, Shuang Wei, Panpan Li, Xianfeng Li

**Affiliations:** Guangzhou Customs District Technology Center, Guangzhou, China

**Keywords:** Mitochondrial genome, *Dynastes satanas*, phylogeny

## Abstract

In this study, we sequenced and analyzed the mitochondrial genome of the Satanas beetle, *Dynastes satanas* Moser, 1909, which was intercepted by Chinese Customs during an attempted smuggling operation in 2022. The complete mitochondrial genome is 16,973 bp in length (GenBank accession number: OQ998898) and contains 13 protein-coding genes, 22 tRNAs, 2 rRNAs, and a control region of 2285 bp. The gene order of trnQ-trnI-trnM in the mitochondrial genome of *Dynastes satanas* is consistent with that of other species in the genus *Dynastes*. All 13 PCGs are initiated by the ATN start codon. Seven genes terminate with the TAA stop codon, one with TAG, and five with a single T. The nucleotide composition of mitochondrial genome of *Dynastes satanas* was 38.07% of A, 31.49% of T, 20.52% of C, and 9.92% of G. Phylogenetic analysis indicates that *Dynastes satanas* and *Dynastes neptunus* (Quensel, 1805) exhibit a considerable genetic distance, suggesting that they should be classified as two distinct subgenera.

## 1. Introductoin

*Dynastes satanas* Moser, 1909, commonly known as the Satanas beetle, belongs to the order Coleoptera, family Scarabaeidae, subfamily Dynastinae, genus *Dynastes*, and the subgenus *Theogenes*. This species is endemic to Bolivia, primarily distributed in the La Paz and Cochabamba provinces (Iannacone-Oliver and Soras-Vega 2010; Dutrillaux and Dutrillaux 2013). In order to protect the Satanas beetle, Bolivia’s proposal to list it in Appendix II of the Convention on International Trade in Endangered Species of Wild Fauna and Flora (CITES) was endorsed during the 15th Conference of the Parties (Goh and Hashim 2019; Crespin and Barahona Segovia 2021; Guerra-Serrudo et al. 2023).

Genus *Dynastes* are significant research subjects within the family of Scarabaeidae, comprising two subgenus: *Dynastes* and *Theogenes*, with a total of 17 reported species. Among the subgenus *Dynastes*, 15 species are categorized into the White Hercules group with 5 species and the Giant Hercules groups with 10 species (Dutrillaux and Dutrillaux 2013; Huang 2017). The subgenus *Theogenes* includes two species: *Dynastes satanas* and *Dynastes neptunus* (Hwang 2011). The classification of this genus relies not only on morphological characteristics but also on geographical distribution and genetic data, making the taxonomic work highly complex and challenging (Huang and Knowles 2016). Therefore, genomic data based on mitochondrial genome contribute to understanding the phylogenetic relationships and evolutionary pathways among species (Cameron et al. 2006). This study enriches the mitochondrial genome database of the genus *Dynastes*, providing valuable information for more accurate reconstruction of interspecies phylogenetic relationships.

## 2. Materials and methods

### 2.1. Sample collection, DNA extraction and sequencing

**Figure 1.**
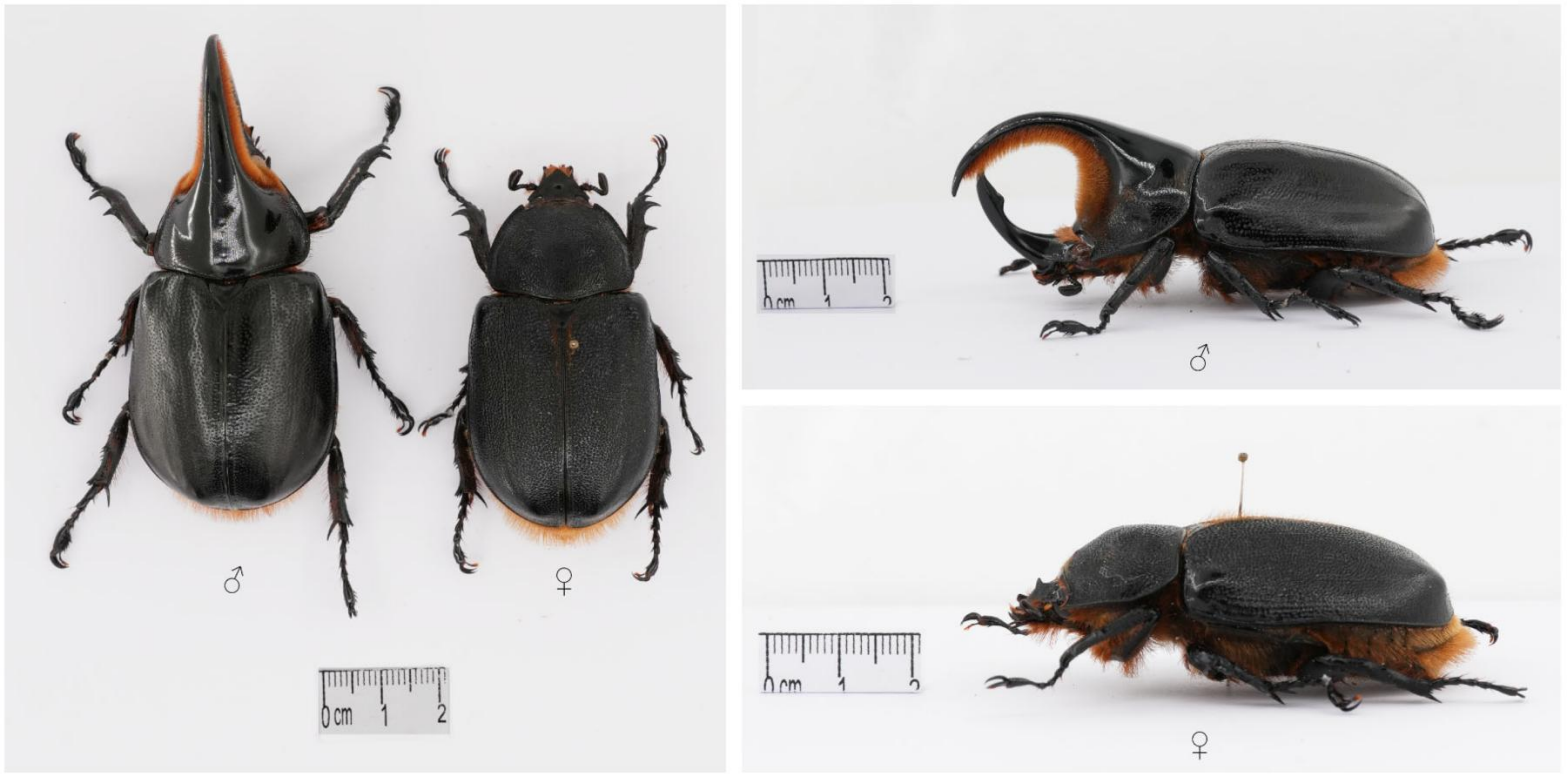
Specimen photographs for male and female adults of *Dynastes satanas*. Some parts of the specimen were damaged and repaired with white latex. Species photos were taken by Xunuo He in Guangzhou Customs District Technology Center, Guangzhou, Guangdong province.

The specimen was intercepted by Guangzhou Customs District (Interception location: 23.1485°N, 113.2535°E) when offenders smuggled it into China through a cross-border parcel from Japan in December 2022 (Figure 1). The specimen was deposited at the Guangzhou Customs District Technology Center, Guangzhou with the voucher number IQTC-PI-22010 (contact: Xunuo He, 522851312@qq.com).

Total genomic DNA was extracted from the muscle tissue of the adult male foreleg tibia using a DNeasy tissue kit (Qiagen, Beijing, China), following the manufacturer’s protocols (Lear et al. 2018). After isolating the DNA, 1 μg sample DNA was fragmented to a size of 300 bp–500 bp using the Covaris M220 system (Sakamoto et al. 2021; Füllgrabe et al. 2023). Short-insert libraries were constructed according to the manufacturer’s instructions (TruSeq^™^ Nano DNA Sample Prep Kit, Illumina) and sequenced on an Illumina NovaSeq 6000 platform (BIOZERON Co., Ltd, Shanghai, China) with 150 bp paired-end reads (Jeon et al. 2021; Modi et al. 2021).

### 2.2. Mitochondrial genome assembly and annotation

We processed the raw data using Trimmomatic (v0.39) to obtain the final high-quality data for subsequent assembly. Using the built-in mitochondrial genome seed database in GetOrganelle (v1.7.5), second-generation sequencing reads were filtered and enriched. We then performed de novo assembly with SPAdes (v3.0.0), producing a standardized circular mitochondrial genome sequence.

The mitochondrial genome was de novo annotated using the online MITOS tool (https://usegalaxy.eu/) with the invertebrate genetic code and default parameters (Bernt et al. 2013). We then manually aligned and refined the preliminary annotations against the reference genome of *Dynastes hercules* (GenBank accession number: OK484309) to accurately determine gene start and stop codon positions, resulting in a highly accurate set of conserved genes (Ayivi et al. 2021; Cigarroa-Toledo et al. 2024). A circular map of the mitochondrial genome was generated using the CGView tool (https://proksee.ca).

### 2.3. Phylogenetic analysis

We reconstructed the phylogenetic tree using 14 mitochondrial genome sequences, including 11 from the genus *Dynastes* and 2 from the genus *Xylotrupes*, both sourced from GenBank, along with 1 sequence newly generated in this study. Due to the absence of annotation files for 10 mitochondrial genome sequences, we utilized Biopython (v1.84) and BLAST+ (v2.16.0) to extract 13 PCGs from these genomes, using the mitochondrial genome sequence of OK484309 as a reference. The newly extracted PCGs were aligned with those from four other genomes using MAFFT (v7.505) for multiple sequence alignment. The aligned sequences were then concatenated for subsequent phylogenetic tree construction.

We used two species from the genus *Xylotrupes* as outgroups. The maximum likelihood (ML) tree was constructed using IQ-TREE (v2.3.6) under the model TPM2u + F+R3, with the bootstrap value set to 1,000,000 to assess branch reliability while also calculating branch length among the species. The phylogenetic tree was visualized using iTOL (https://itol.embl.de).

## 3. Result

**Figure 2.**
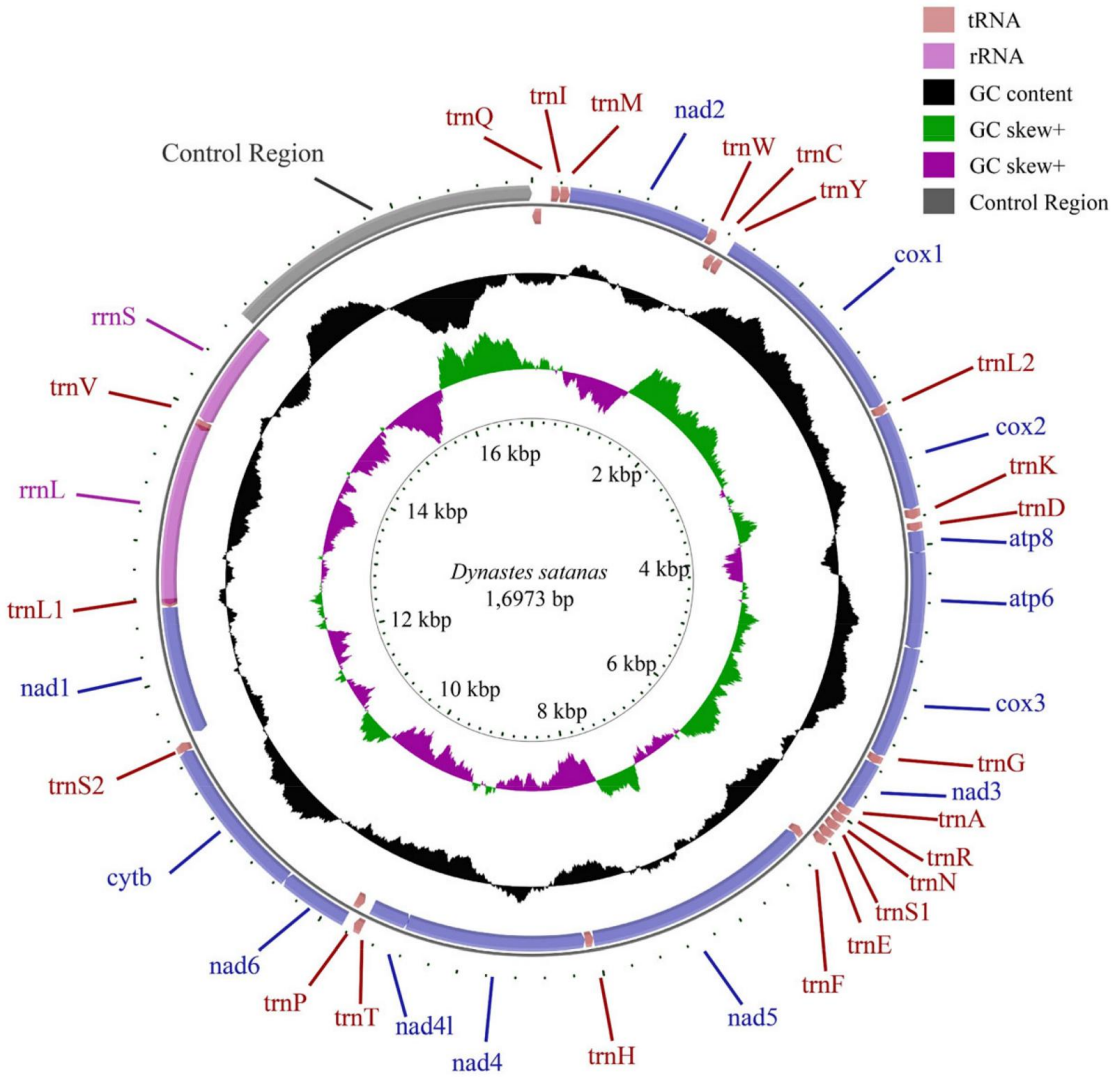
Mitochondrial genome structure of *Dynastes satanas.*

The minimum and maximum read mapping depths for the assembled mitochondrial genome were 72× and 7137×, respectively, with an average depth of 722.86× (Supplementary Figure S1). The initial sequencing data amounted to 7005 MB. After processing, the cleaned data size was reduced to 6993 MB, resulting in a final assembled mitochondrial genome sequence length of 16,793 bp. The mitochondrial genome of *Dynastes satanas* includes 13 PCGs, 22 tRNAs, 1 rrnS gene, 1 rrnL gene, and 2285 bp of control region (Figure 2).

In the mitochondrial genome of *Dynastes satanas*, 9 PCGs and 14 tRNAs are encoded on the N strand, while the remaining 14 genes are encoded on the J strand. Three tRNAs have undergone rearrangement between the control region and *nad2*, with the gene order now being *trnQ*-*trnI*-*trnM*. This rearrangement is consistent with other species within the genus *Dynastes*, differing from the ancestral insect gene order of *trnI*-*trnQ*-*trnM* (Cameron et al. 2006; Shao et al. 2014; Morgan et al. 2022). Additionally, eight intergenic spacers totaling 117 bp and twelve overlapping regions totaling 112 bp are dispersed throughout the genome (Supplementary Table S1).

All 13 PCGs are initiated by the ATN start codon, with 4 using ATT, 4 using ATC, and 5 using ATG. Seven genes terminate with the TAA stop codon, one with TAG, and five with a single T. The lengths of the *rrnL* and *rrnS* genes are 1365 bp and 790 bp, respectively, with A + T contents of 73.03% and 71.65%, respectively. Both the *rrnL* and *rrnS* exhibit a negative AT skew and a positive GC skew, indicating a higher proportion of T and G compared to A and C. This demonstrates a bias toward T and G bases in the rRNAs.

**Figure 3.**
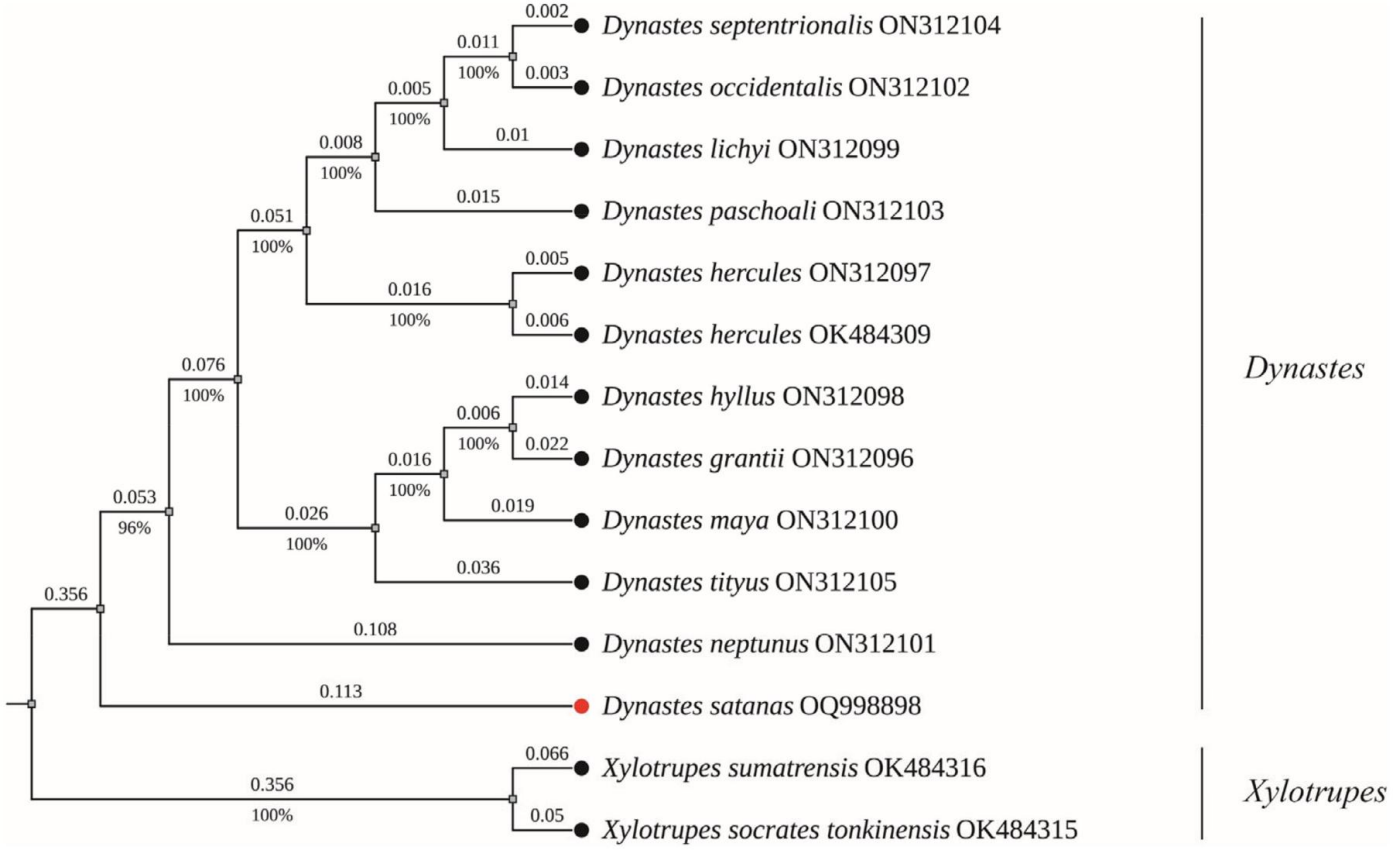
Phylogenetic tree constructed using the ML method based on sequences from 13 PCGs of the mitochondrial genome. Branch lengths are indicated above the branches, with bootstrap support values below. All species in the tree are labeled with their scientific names and GenBank accession numbers on the right side. The position of *Dynastes satanas* is highlighted by a red circle. The sequences used are as follows: *Dynastes septentrionalis* ON312104, *Dynastes occidentalis* ON312102, *Dynastes lichyi* ON312099, *Dynastes paschoali* ON312103, *Dynastes hercules* ON312097, *Dynastes hyllus* ON312098, *Dynastes grantii* ON312096, *Dynastes maya* ON312100, *Dynastes tityus* ON312105, *Dynastes neptunus* ON312101 (Morgan et al. 2022); *Dynastes hercules* OK484309, *Xylotrupes sumatrensis* OK484316, and *Xylotrupes socrates tonkinensis* OK484315 (Ayivi et al. 2021).

The phylogenetic analysis reveals that *Dynastes granti*, *Dynastes hyllus*, *Dynastes maya*, and *Dynastes tityus* form a distinct clade, while *Dynastes septentrionalis*, *Dynastes occidentalis*, *Dynastes lichyi*, and *Dynastes paschoali* form another well-supported clade (Figure 3). These two clades combine into a larger clade, consistent with prior studies that classify them as the White Hercules group and the Giant Hercules group (Huang and Knowles 2016). Additionally, the analysis indicates that *Dynastes satanas* and *Dynastes neptunus* exhibit a considerable genetic distance (branch length: 0.27), which exceeds the distance between *Dynastes neptunus* and *Dynastes tityus* (branch length: 0.24) (Supplementary Table S2).

## 4. Discussion and conclusions

In this study, we reported the complete mitochondrial genome sequence of *Dynastes satanas*, which has a total length of 16,973 bp, including 37 genes and a control region. Its gene order is consistent with the published mitochondrial genomes of the genus *Dynastes*. Due to the limitations of Illumina sequencing, we were only able to sequence a portion of the control region, necessitating the use of more advanced sequencing technologies for further investigation (Morgan et al. 2022). Additionally, the GC content of the 13 PCGs in *Dynastes satanas* is lower than that of *Dynastes hercules*, averaging 5.58% less, which may be related to the environmental selection in the specific region of Bolivia where *Dynastes satanas* is distributed (Cameron et al. 2006; Iannacone-Oliver and Soras-Vega 2010).

In all insects of the subfamily Dynastinae, mitochondrial genome rearrangements have occurred. This rearrangement is characterized by the gene order of *trnQ*-*trnI*-*trnM*, which differs from the more common *trnI*-*trnQ*-*trnM* found in most scarab beetles (Shao et al. 2014; Ayivi et al. 2021; Mello et al. 2021). This gene order is considered a characteristic or a synapomorphy of this subfamily, and the gene arrangement in *Dynastes satanas* is consistent with this phenomenon.

According to recent research, Huang has elevated 10 species within the subgenus *Dynastes* from subspecies to species-level classification (Keller and Cave 2016; Huang 2017, 2016). However, our phylogenetic analysis indicates that the branch lengths among these elevated species contradict established criteria for species classification. Furthermore, the genetic distance between *Dynastes satanas* and *Dynastes neptunus* is considerable, suggesting that these two species should be classified into different subgenera rather than within the current subgenus *Theogenes*. Nonetheless, due to challenges in obtaining specimens and the limited mitochondrial genome data available, these conclusions require further data and evidence for validation.

## Supplementary Material

Supplementary_Figure S1_Table S1_Table S2.docx

## Data Availability

The genome sequence data that support the findings of this study are openly available in GenBank of NCBI (https://www.ncbi.nlm.nih.gov/nuccore/OQ998898) under the accession no. OQ998898. The associated **BioProject**, **SRA**, and **Bio-Sample** numbers are, PRJNA1133938, SRR29778415, and SAMN42391249, respectively.
