# Supplementary material for: Complete mitochondrial genome of the Satanas beetle, Dynastes satanas Moser, 1909 (Coleoptera: Scarabaeidae)
Source: Mitochondrial DNA B Resour. 2024 Dec 1;9(12):1627–31. doi: 10.1080/23802359.2024.2432373 (PMC11613335; doi:10.1080/23802359.2024.2432373)
Supplement: Supplementary_Figure S1_Table S1_Table S2.docx [file TMDN_A_2432373_SM8149.docx]

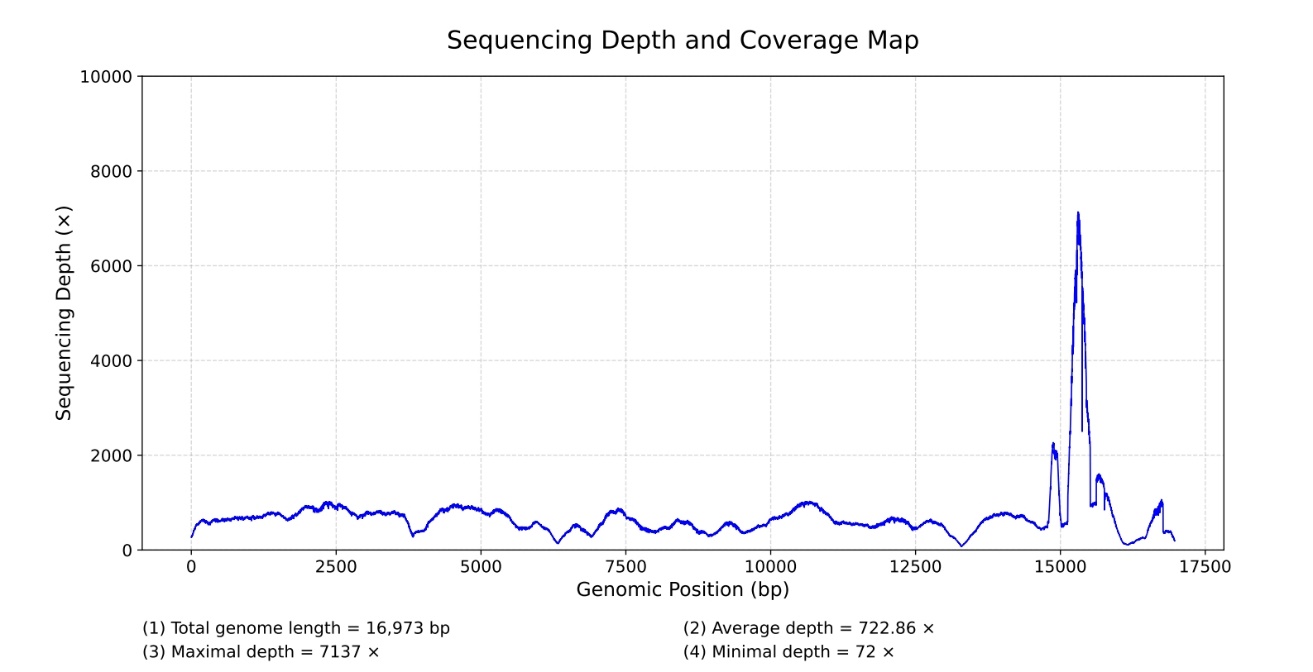


Figure S1. The estimated coverage depths from Illuminasequencing reads for *Dynastes satanas* mitogenome

Table S1. Annotation table of *Dynastes satanas* mitogenome.

| Gene | Strand | Position(Start-End) | Length(bp) | Intergenic_spacer | Start_coden | Stop_coden |
| --- | --- | --- | --- | --- | --- | --- |
| trnQ | R | 1-67 | 67 | - | - | - |
| trnI | F | 135-198 | 64 | 67 | - | - |
| trnM | F | 199-266 | 68 | 0 | - | - |
| nad2 | F | 267-1274 | 1008 | 0 | ATT | TAA |
| trnW | F | 1274-1339 | 66 | -1 | - | - |
| trnC | R | 1332-1396 | 65 | -8 | - | - |
| trnY | R | 1397-1460 | 64 | 0 | - | - |
| cox1 | F | 1462-2992 | 1531 | 1 | ATC | T |
| trnL2 | F | 2993-3058 | 66 | 0 | - | - |
| cox2 | F | 3059-3746 | 688 | 0 | ATC | T |
| trnK | F | 3747-3817 | 71 | 0 | - | - |
| trnD | F | 3842-3903 | 62 | 24 | - | - |
| atp8 | F | 3904-4059 | 156 | 0 | ATT | TAA |
| atp6 | F | 4053-4724 | 672 | -7 | ATG | TAA |
| cox3 | F | 4724-5510 | 787 | -1 | ATG | T |
| trnG | F | 5511-5574 | 64 | 0 | - | - |
| nad3 | F | 5575-5926 | 352 | 0 | ATT | T |
| trnA | F | 5927-5991 | 65 | 0 | - | - |
| trnR | F | 5992-6055 | 64 | 0 | - | - |
| trnN | F | 6058-6122 | 65 | 2 | - | - |
| trnS1 | F | 6123-6189 | 67 | 0 | - | - |
| trnE | F | 6190-6252 | 63 | 0 | - | - |
| trnF | R | 6251-6314 | 64 | -2 | - | - |
| nad5 | R | 6315-8028 | 1714 | 0 | ATT | T |
| trnH | R | 8029-8092 | 64 | 0 | - | - |
| nad4 | R | 8092-9429 | 1338 | -1 | ATG | TAA |
| nad4l | R | 9423-9713 | 291 | -7 | ATG | TAA |
| trnT | F | 9716-9780 | 65 | 2 | - | - |
| trnP | R | 9781-9846 | 66 | 0 | - | - |
| nad6 | F | 9848-10351 | 504 | 1 | ATC | TAA |
| cob | F | 10351-11493 | 1143 | -1 | ATG | TAG |
| trnS2 | F | 11492-11556 | 65 | -2 | - | - |
| nad1 | R | 11576-12526 | 951 | 19 | ATC | TAA |
| trnL1 | R | 12528-12590 | 63 | 1 | - | - |
| rrnL | R | 12546-13910 | 1365 | -45 | - | - |
| trnV | R | 13875-13944 | 70 | -36 | - | - |
| rrnS | R | 13944-14733 | 790 | -1 | - | - |
| CR | - | 14734-16973 | 2285 | 0 | - | - |

| accession No. | OK484309 | OK484315 | OK484316 | OQ998898 | ON312101 | ON312096 | ON312098 | ON312100 | ON312105 | ON312099 | ON312102 | ON312104 | ON312103 | ON312097 |
| --- | --- | --- | --- | --- | --- | --- | --- | --- | --- | --- | --- | --- | --- | --- |
| OK484309 | 0.00 | 0.96 | 0.98 | 0.31 | 0.26 | 0.14 | 0.13 | 0.13 | 0.13 | 0.04 | 0.05 | 0.05 | 0.04 | 0.01 |
| OK484315 | 0.96 | 0.00 | 0.12 | 0.88 | 0.92 | 0.96 | 0.95 | 0.95 | 0.95 | 0.96 | 0.97 | 0.97 | 0.96 | 0.96 |
| OK484316 | 0.98 | 0.12 | 0.00 | 0.89 | 0.94 | 0.98 | 0.97 | 0.97 | 0.97 | 0.98 | 0.98 | 0.98 | 0.98 | 0.98 |
| OQ998898 | 0.31 | 0.88 | 0.89 | 0.00 | 0.27 | 0.31 | 0.30 | 0.30 | 0.30 | 0.31 | 0.32 | 0.32 | 0.31 | 0.31 |
| ON312101 | 0.26 | 0.92 | 0.94 | 0.27 | 0.00 | 0.25 | 0.25 | 0.24 | 0.24 | 0.26 | 0.26 | 0.26 | 0.26 | 0.25 |
| ON312096 | 0.14 | 0.96 | 0.98 | 0.31 | 0.25 | 0.00 | 0.04 | 0.05 | 0.08 | 0.14 | 0.15 | 0.15 | 0.14 | 0.14 |
| ON312098 | 0.13 | 0.95 | 0.97 | 0.30 | 0.25 | 0.04 | 0.00 | 0.04 | 0.07 | 0.13 | 0.14 | 0.14 | 0.13 | 0.13 |
| ON312100 | 0.13 | 0.95 | 0.97 | 0.30 | 0.24 | 0.05 | 0.04 | 0.00 | 0.07 | 0.13 | 0.14 | 0.14 | 0.13 | 0.13 |
| ON312105 | 0.13 | 0.95 | 0.97 | 0.30 | 0.24 | 0.08 | 0.07 | 0.07 | 0.00 | 0.13 | 0.14 | 0.14 | 0.13 | 0.13 |
| ON312099 | 0.04 | 0.96 | 0.98 | 0.31 | 0.26 | 0.14 | 0.13 | 0.13 | 0.13 | 0.00 | 0.02 | 0.02 | 0.03 | 0.04 |
| ON312102 | 0.05 | 0.97 | 0.98 | 0.32 | 0.26 | 0.15 | 0.14 | 0.14 | 0.14 | 0.02 | 0.00 | 0.00 | 0.03 | 0.05 |
| ON312104 | 0.05 | 0.97 | 0.98 | 0.32 | 0.26 | 0.15 | 0.14 | 0.14 | 0.14 | 0.02 | 0.00 | 0.00 | 0.03 | 0.05 |
| ON312103 | 0.04 | 0.96 | 0.98 | 0.31 | 0.26 | 0.14 | 0.13 | 0.13 | 0.13 | 0.03 | 0.03 | 0.03 | 0.00 | 0.04 |
| ON312097 | 0.01 | 0.96 | 0.98 | 0.31 | 0.25 | 0.14 | 0.13 | 0.13 | 0.13 | 0.04 | 0.05 | 0.05 | 0.04 | 0.00 |

Table S2. Phylogenetic branch length matrix using the ML method based on sequences from 13 protein-coding genes of the mitochondrial genome.
